# Herpes Simplex Virus-2 Meningitis Masquerading as Pseudotumor Cerebri

**DOI:** 10.7759/cureus.15764

**Published:** 2021-06-19

**Authors:** Robin Sherchan, Jishna Shrestha, Yetunde B Omotosho, Nataliia Dyatlova, Jenie S Nepomuceno

**Affiliations:** 1 Internal Medicine, Northwestern Medicine McHenry Hospital, Rosalind Franklin University of Medicine and Science, McHenry, USA; 2 Internal Medicine, Northwestern Medicine McHenry Hospital, Metro Infectious Disease Consultants, McHenry, USA

**Keywords:** aseptic meningitis, hsv-2, pseudotumor cerebri, idiopathic intracranial hypertension, intracranial hypertension secondary to viral infection, papilledema, herpes simplex virus, hsv meningitis, elevated intracranial pressure, viral meningitis causing elevated icp

## Abstract

We report a case of a 27-year-old obese female presenting with headache, blurry and double vision. She was found to have bilateral papilledema by an ophthalmologist and sent to the emergency department (ED). Cerebrospinal fluid (CSF) analysis showed elevated opening pressure and lymphocytic pleocytosis. Symptoms improved significantly after lumbar puncture (LP). Subsequently, polymerase chain reaction (PCR) for herpes simplex virus-2 (HSV-2) came back positive. This case represents an unusual presentation of HSV-2 meningitis, where the clinical picture was suggestive of pseudotumor cerebri or idiopathic intracranial hypertension (IIH), but CSF analysis revealed HSV-2. Papilledema and elevated intracranial pressure has not previously been described in association with HSV-2. Therefore, patients presenting with typical signs and meeting all diagnostic criteria for IIH in the presence of CSF pleocytosis may represent a distinct group of viral-induced intracranial hypertension. In these cases, an investigation of viral etiologies should be conducted.

## Introduction

Pseudotumor cerebri or idiopathic intracranial hypertension (IIH) is diagnosed in the presence of elevated intracranial pressure (cerebrospinal fluid (CSF) pressure > 25 cm H2O), normal CSF composition in conjunction with normal radiographic imaging [1,2]. The most common presenting symptoms include headache, visual disturbances most commonly diplopia (related to abducens nerve palsy), and pulsatile tinnitus [1]. One of the essential criteria for IIH is normal CSF composition. There have been reports of CSF showing pleocytosis where the clinical picture is suggestive of IIH [3]. 

Primary herpes simplex virus-2 (HSV-2) infection occurs as skin or mucosal lesion. Many of the patients are unaware of their HSV-2 exposure. Subsequently, retrograde transport of the virus to the peripheral sensory ganglia occurs, after which the virus can reactivate periodically with antegrade transmission to the nerve endings. HSV-2 remains latent in peripheral sensory ganglia and can persist lifelong in the host. Latent HSV infection is reactivated by local and systemic stimuli, such as illness or other factors that may cause reactivation [4,5]. HSV-2 is classically associated with recurrent genital herpes [6] and aseptic meningitis [5]. It has also been determined as one of the causes of Mollaret syndrome (a recurrent self-limiting aseptic meningitis) [5,7]. 

We report a case of confirmed HSV-2 meningitis with typical signs that meet all diagnostic criteria for IIH except a normal CSF report in an immunocompetent patient. Intracranial hypertension secondary to viral infection has been described in cases of varicella-zoster virus (VZV), Epstein-Barr virus (EBV), human herpesvirus 6 (HHV-6), measles, enterovirus, and human immunodeficiency virus (HIV) [3,4,8]. HSV-2 presenting with elevated intracranial pressure has not been reported to the best of our knowledge.

This article was previously submitted as a case report for IDWeek 2021 conference and may be published on the ID Images website if it is accepted for IDWeek 2021 Conference.

## Case presentation

A 27-year-old obese woman with no significant past medical history presented to the emergency department (ED) for headache, blurry and double vision. Earlier in the day, she was seen by an ophthalmologist in the office who noted bilateral papilledema and urged the patient to go to the ED immediately. The diplopia and blurry vision started two weeks prior to presentation and were initially intermittent; however, progressively worsened to be constant. She described the blurry vision more in the peripheral visual field and double vision, worse when looking to the far right side. The patient is a travel blogger and spent most of her time in Indonesia last year. She recently went scuba diving in Mexico six weeks prior to the presentation. She did two dives around 30 feet, each lasting approximately 45 minutes. Subsequently, after the dive, the patient had headaches. She was treated with levofloxacin for a possible eardrum injury. She traveled back to the United States five days later, but she continued to have headaches with mild photosensitivity. She started biweekly hyperbaric treatment as an outpatient, which helped with the headache. During this time, she also reported occasional episodes of tinnitus. 

**Figure 1:**
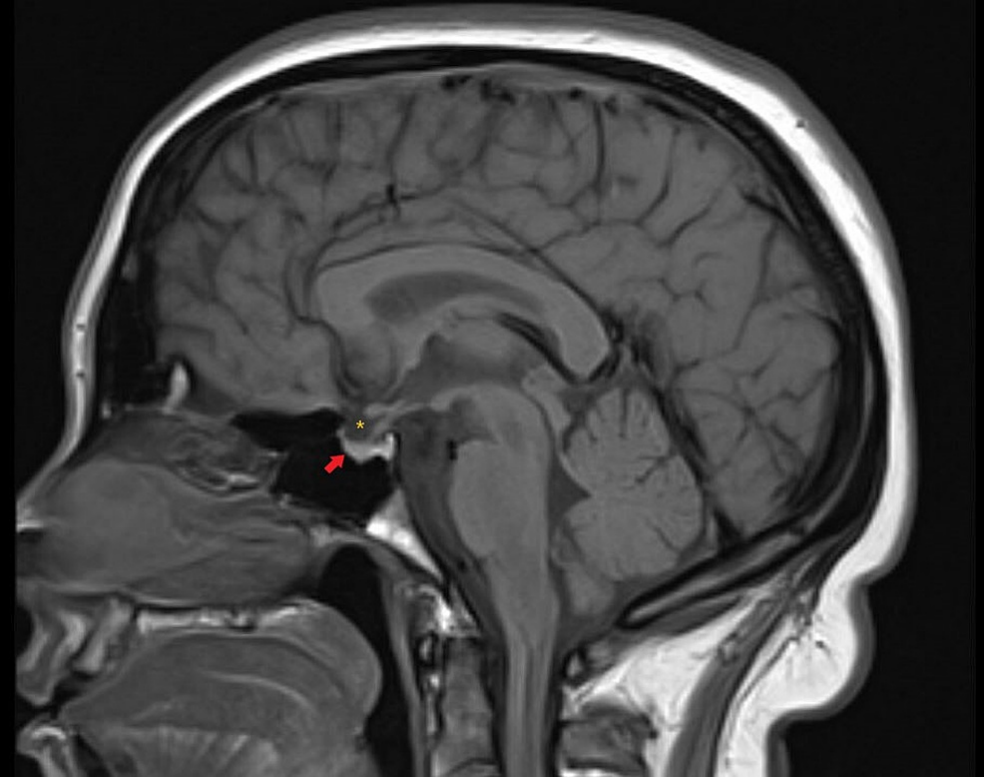
Magnetic resonance imaging brain: Sagittal T1 weighted image showing a flattened pituitary gland (red arrow) suggestive of partially empty sella appearance and cerebrospinal fluid in the sella (yellow asterisk)

**Figure 2:**
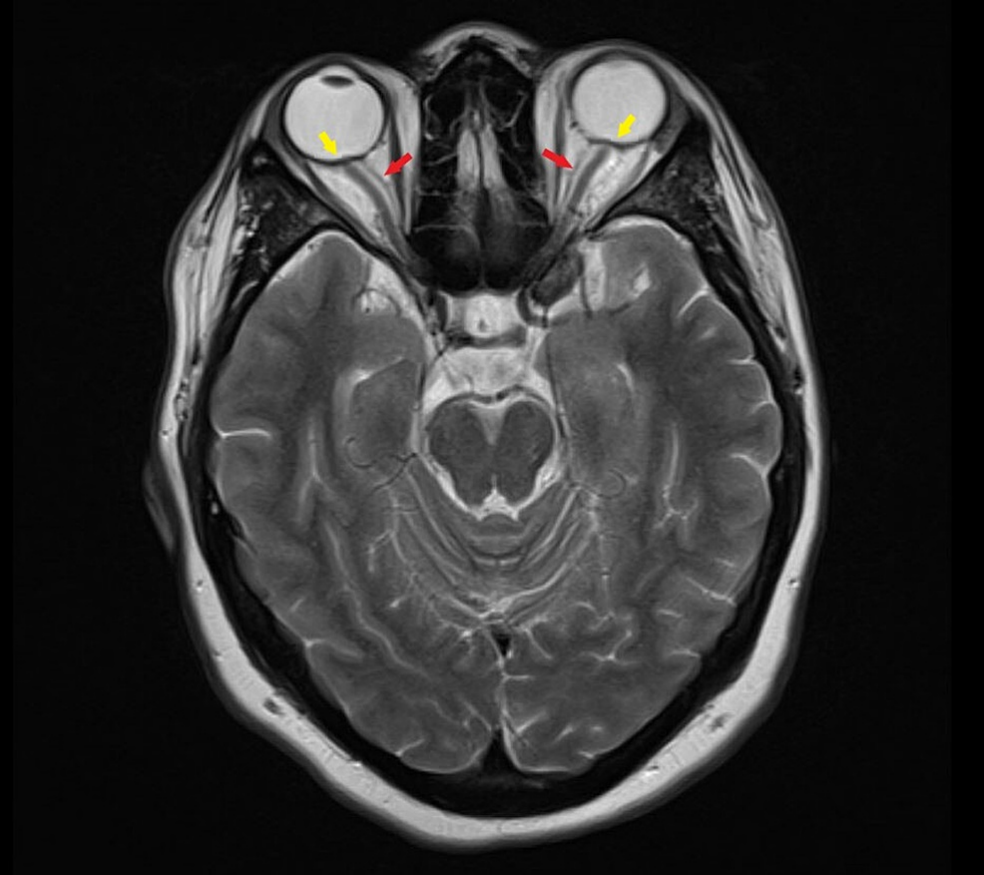
Magnetic resonance imaging brain: Axial T2 weighted image at the level of the optic nerve showing hyperintense fluid (cerebrospinal fluid) around the bilateral optic nerves (red arrows) and bulging of bilateral optic nerve papillae suggestive of papilledema (yellow arrow)

On physical examination, she was alert, oriented, and cooperative. Vital signs were normal. Her body mass index (BMI) was 31.1 kg/m^2^. Extraocular movement exam revealed right lateral rectus palsy with a subjective complaint of double vision on the far right gaze. Other extraocular movements and pupillary responses were normal. Meningeal signs were negative. The ear exam, along with the remainder of the neurological exam, was normal. Initial labs showed mild leukocytosis of 11700/uL with neutrophils of 69% and lymphocytes of 24.9%, C-reactive protein was mildly elevated. Other labs, including electrolytes, renal function test, liver function test, and erythrocyte sedimentation rate were normal. Computerized Tomography (CT) of the head, CT angiography of the head and neck, and magnetic resonance venography (MRV) were unremarkable. Magnetic resonance imaging (MRI) of the head showed a flattened pituitary gland fluid around the bilateral optic nerves, and bulging of bilateral optic nerve papillae suggestive of papilledema, which could be seen in IIH (Figures 1-2)

Lumbar puncture (LP) was performed which revealed an opening pressure of 52 cm of H_2_O. CSF analysis showed an elevated white cell count of 208/uL with a lymphocytic predominance of 89%. CSF glucose was low at 33 mg/dL (serum glucose 73 mg/dL) and protein was elevated at 76 mg/dL. The patient was empirically started on ceftriaxone and vancomycin. Ceftriaxone was switched to meropenem by infectious disease to cover possible pseudomonas infection causing an ear infection/petrous apicitis. Her headache improved after LP, but the patient still had horizontal diplopia. Acetazolamide 500 mg twice daily was initiated. Two days after the initial LP, a second LP was performed, showing an opening pressure of 31 cm of H_2_O. CSF WBC was now 169/uL with a lymphocyte count of 89%. On further questioning, the patient mentioned she had an episode of genital HSV infection eight months back. At this point, acyclovir was started empirically. Transfer to tertiary center was initiated for tropical infectious disease input (given travel history) and uncertain etiology of her presentation. After transfer, acetazolamide dosage was increased to 1000 mg twice daily. 

CSF cultures, including fungal culture, were negative. The patient had significant improvement in her symptoms and as the cultures were negative, antibiotics were discontinued. CSF HSV-2 polymerase chain reaction (PCR) came back positive. She was discharged on acetazolamide 1000 mg twice daily and oral valacyclovir for two weeks. On one month follow-up with an ophthalmologist, the patient no longer had headaches or vision issues. The papilledema had resolved entirely, and acetazolamide taper was initiated.

Differential diagnosis

For this case, various differential diagnoses were considered. Symptoms started after diving, therefore, the possibility of barotrauma and decompression sickness was entertained. Previously decompression sickness has been thought to occur in dives more than 30 feet, but there are cases where it has been seen in a shallow water dive [9]. Although the patient might have had some barotrauma-related symptoms associated with diving, on presentation to ED, this was ruled out based on the duration of symptoms, exam, imaging, and CSF findings [10]. Gradenigo syndrome (triad of petrous apicitis, abducens nerve palsy, retro-orbital pain) was ruled out based on MRI showing normal middle ear and temporal bone with normal petrous apices [11]. Other differentials included fungal meningitis, viral meningitis, tubercular meningitis, Lyme disease, sarcoidosis, and autoimmune diseases. Therefore, CSF studies for VZV, EBV, cytomegalovirus (CMV), Cryptococcus, Histoplasma, Coccidioides, Mycobacterium species, syphilis, and Lyme disease were sent and eventually came back negative. Blood tests for West Nile virus, Chikungunya, Zika virus, syphilis, HIV, Interferon-gamma (IFN-γ) release assay for tuberculosis were also negative. Antinuclear antibody, complement C3 and C4 levels, and angiotensin-converting enzyme levels were normal.

## Discussion

HSV-2 neurologic manifestation may result from primary infection or reactivation of latent HSV-2 [5]. HSV-2 aseptic meningitis is prevalent among women compared to men with HSV-2 genital infection [5] though cases without genital infection have been described [12]. HSV-2 CSF analysis is usually lymphocyte-predominant with normal glucose and normal CSF pressure [13]. There are cases reported where low CSF glucose (hypoglycorrhachia) is seen with glucose less than fifty percent of normal blood glucose levels in patients with viral meningitis due to herpes simplex, mumps, and lymphocytic choriomeningitis [14]. PCR is the preferred diagnostic test for suspected HSV-associated meningitis based on studies conducted in patients with HSV encephalitis. The exact sensitivity and specificity of PCR for HSV-2 meningitis are not known [15]. Viral culture has only around 50 percent sensitivity as DNA levels are generally lower among patients with clinical meningitis than encephalitis [13]. In some patients, active or a history of genital lesions may be absent. In such cases, if the CSF picture is suspicious, then the HSV PCR test is still warranted [12]. 

The modified Dandy criteria [16] for diagnosis of pseudotumor cerebri include: (1) symptoms and signs of increased intracranial pressure (headache, nausea, vomiting, transient obscuration of vision, papilledema); (2) normal neurological examination except unilateral or bilateral abducens nerve palsy; (3) absence of a mass lesion or enlarged ventricles; and (4) increased CSF opening pressure > 25 cm H_2_O, with normal CSF analysis. Our patient had headaches, tinnitus, and visual symptoms including diplopia related to abducens nerve palsy along with papilledema and elevated intracranial pressure. Patients who present with typical signs and meet all diagnostic criteria for IIH other than a normal CSF report may represent a distinct group of intracranial hypertension secondary to viral infection (IHSVI). The presence of characteristic CSF showing pleocytosis with lymphocytic predominance should prompt us to think of viral etiologies of elevated intracranial pressure [3,4]. With numerous cases reported showing a possible association between intracranial hypertension and viral meningitis, other causative organisms for intracranial hypertension may be identified with the advent of more advanced testing. HSV-2 causing meningitis often has a self-limiting course in immunocompetent patients and often resolves without specific therapy. Treatment of immunocompetent hosts with antivirals is still unclear, although there is a definite benefit in preventing neurologic sequelae in immunocompromised patients [17]. As such, antiviral initiation should be based on the clinical picture.

## Conclusions

Several case reports have demonstrated elevated intracranial pressure in the setting of underlying viral meningitis. Some of the viruses isolated are VZV, EBV, HHV-6, enterovirus, measles, and HIV. HSV-2 meningitis presenting with elevated pressure has not been reported in the literature to date to the best of our knowledge. In the setting of raised intracranial pressure and CSF lymphocytosis, viral etiologies of elevated intracranial pressure should be looked into before coining it as IIH, and viral PCR tests should be considered.

## References

[1] Friedman DI (2014). The pseudotumor cerebri syndrome. Neurol Clin.

[2] Spennato P, Ruggiero C, Parlato RS, Buonocore MC, Varone A, Cianciulli E, Cinalli G (2011). Pseudotumor cerebri. Childs Nerv Syst.

[3] Ravid S, Shachor-Meyouhas Y, Shahar E, Kra-Oz Z, Kassis I (2013). Viral-induced intracranial hypertension mimicking pseudotumor cerebri. Pediatr Neurol.

[4] Kiefer L, Adam D, Mudugal D, Burnett MS (2020). Viral meningitis mimicking benign intracranial hypertension: a report of two cases. Interdiscip Neurosurg.

[5] Berger JR, Houff S (2008). Neurological complications of herpes simplex virus type 2 infection. Arch Neurol.

[6] Xu F, Sternberg MR, Kottiri BJ (2006). Trends in herpes simplex virus type 1 and type 2 seroprevalence in the United States. JAMA.

[7] Cohen BA, Rowley AH, Long CM (1994). Herpes simplex type 2 in a patient with Mollaret's meningitis: demonstration by polymerase chain reaction. Ann Neurol.

[8] Beal JC (2017). Increased intracranial pressure in the setting of Enterovirus and other viral meningitides. Neurol Res Int.

[9] (2021). Shallow water bends. https://www.undercurrent.org/UCnow/dive_magazine/1998/ShallowWaterBends199810.html.

[10] Pollock NW, Buteau D (2017). Updates in Decompression Illness. Emerg Med Clin North Am.

[11] Jackler RK, Parker DA (1992). Radiographic differential diagnosis of petrous apex lesions. Am J Otol.

[12] O'Sullivan CE, Aksamit AJ, Harrington JR, Harmsen WS, Mitchell PS, Patel R (2003). Clinical spectrum and laboratory characteristics associated with detection of herpes simplex virus DNA in cerebrospinal fluid. Mayo Clin Proc.

[13] Kupila L, Vuorinen T, Vainionpää R, Hukkanen V, Marttila RJ, Kotilainen P (2006). Etiology of aseptic meningitis and encephalitis in an adult population. Neurology.

[14] Ratzan KR (1985). Viral meningitis. Med Clin North Am.

[15] Murphy RF, Caliendo AM (2009). Relative quantity of cerebrospinal fluid herpes simplex virus DNA in adult cases of encephalitis and meningitis. Am J Clin Pathol.

[16] Smith JL (1985). Whence pseudotumor cerebri?. J Clin Neuroophthalmol.

[17] Noska A, Kyrillos R, Hansen G, Hirigoyen D, Williams DN (2015). The role of antiviral therapy in immunocompromised patients with herpes simplex virus meningitis. Clin Infect Dis.

